# Supplementary material for: Prevalence of Hepatitis C viral infection in Ghana: A systematic review and meta-analysis protocol
Source: PLoS One. 2025 Apr 16;20(4):e0321483. doi: 10.1371/journal.pone.0321483 (PMC12002434; doi:10.1371/journal.pone.0321483)
Supplement: S2 File — (DOCX) [file pone.0321483.s002.docx]

**JOANNA BRIGG’S INSTITUTE APPRAISAL CHECKLIST**

Reviewer_____________________________________Date_____________________________

Author_____________________________________Year_________ Record Number_________

Yes No Unclear Not

applicable

1. Were the criteria for inclusion in the sample clearly

defined?

2. Were the study subjects and the setting described in

detail?

3. Was the exposure measured in a valid and reliable

way?

4. Were objective, standard criteria used for

measurement of the condition?

5. Were confounding factors identified?

6. Were strategies to deal with confounding factors

stated?

7. Were the outcomes measured in a valid and reliable

way?

8. Was appropriate statistical analysis used?

Overall appraisal: Include

Exclude

Seek further info

Comments (Including reason for exclusion)

______________________________________________________________________________
